# Commonly Prescribed Chronic Pharmacological Medications as Risk Factors for Breast Cancer-Related Lymphedema: An Observational Retrospective Cohort Study

**DOI:** 10.3390/healthcare13070691

**Published:** 2025-03-21

**Authors:** Margherita B. Borg, Marco Battaglia, Laura Mittino, Alberto Loro, Laura Lanzotti, Lorenza Scotti, Giuseppina Gambaro, Marco Invernizzi, Alessio Baricich

**Affiliations:** 1 Physical and Rehabilitation Medicine, Department of Health Sciences, University of Eastern Piedmont, 28100 Novara, Italy; marco.battaglia@uniupo.it (M.B.); 20008867@studenti.uniupo.it (L.M.); 20042324@studenti.uniupo.it (A.L.); marco.invernizzi@med.uniupo.it (M.I.); 2 Physical and Rehabilitation Medicine Unit, Azienda Ospedaliero-Universitaria “Maggiore della Carità”, 28100 Novara, Italy; laura.lanzotti@maggioreosp.novara.it; 3 Department of Translational Medicine, University of Eastern Piedmont, 28100 Novara, Italy; lorenza.scotti@uniupo.it; 4 LILT Novara ONLUS Association, 28100 Novara, Italy; giuse55gambaro@gmail.com; 5 Dipartimento Attività Integrate Ricerca e Innovazione (DAIRI), Translational Medicine, Azienda Ospedaliera SS. Antonio e Biagio e Cesare Arrigo, 15121 Alessandria, Italy; 6 Department of Biomedical Sciences, Humanitas University, 20072 Pieve Emanuele, Italy; alessio.baricich@hunimed.eu; 7 Rehabilitation Unit, IRCSS Humanitas Research Hospital, 20089 Rozzano, Italy

**Keywords:** breast cancer-related lymphedema, risk factors, breast cancer, rehabilitation

## Abstract

**Background/Objectives**: Breast cancer-related lymphedema (BCRL) is a significant complication of breast cancer (BC) treatment, characterized by swelling and fluid accumulation. Many risk factors have already been proven to be related to BCRL; meanwhile, many others are still debated and poorly investigated in the literature. This study investigated the potential involvement of commonly prescribed chronic medications in BCRL development. **Methods**: This observational retrospective cohort study included 162 post-surgical breast cancer survivors attending an oncological rehabilitation outpatient service between January 2021 and April 2023. BCRL was diagnosed by physicians through clinical evaluation and objective measures (≥2 cm increase in circumferential girth measurements). Descriptive statistics summarized patient characteristics, and Cox regression models (univariable and multivariable) were employed to analyze risk factors for BCRL. **Results**: BCRL was observed in 53% of participants. The univariable model identified BMI (hazard ratio 1.07, 95% CI 1.02–1.11), overweight (BMI ≥ 25) (HR 1.46, 95% CI 0.95–2.25), and breast prosthesis implantation (HR 1.75, 95% CI 1.09–2.80) as potential risk factors for lymphedema. In the multivariable model, overweight (HR 2.90, 95% CI 1.18–7.14), hypertension (HR 5.09, 95% CI 1.88–13.79), radiotherapy (HR 3.67, 95% CI 1.43–9.38), and breast prosthesis implantation (HR 8.93, 95% CI 2.77–28.81) were identified as independent risk factors for BCRL. **Conclusions**: The findings emphasize the need for further research to understand the role of chronic medications in BCRL risk comprehensively.

## 1. Introduction

Breast cancer (BC) represents the most prevalent female malignant tumor, with an estimated 2.3 million new cases and 685,000 deaths in 2020 worldwide. This pathology constitutes the leading cause of death among young women, especially in less developed countries around the world [1,2,3]. In recent years, novel surgical and non-surgical treatments have led to a constant increase in BC prognosis and a remarkable decrease in therapy-related adverse events. Consequently, despite its high prevalence and incidence, we’ve reached outstanding values of 90% for a 5-year survival rate and 80% for a 10-year survival [4]. Therefore, preserving the health-related quality of life (HR-QoL) and the general well-being of BC survivors is crucial in medicine [5].

About 20% of BC survivors develop post-treatment breast cancer-related lymphedema (BCRL), which is an excess accumulation of protein-rich fluid in the affected side [1,2]. BCRL not only is an aesthetic issue but also brings many limitations and potential complications, such as pain, heaviness, recurrent skin infections, decreased upper-limb range of motion, and affected motor skills (both gross and fine) [3]. All these dysfunctions lead to impaired daily function and a decreased quality of life [4,5,6].

Given the vast incidence of BCRL worldwide and its chronic and recurrent disease characteristics, the literature shows many studies about possible treatment protocols and techniques [3,5,7,8,9]. The first goal of all these protocols is to correctly instruct the patient on what to do and not do. There are many guidelines on BCRL daily care, which all agree on maintaining good skin hygiene (avoiding skin cracks), avoiding any kind of trauma (including venipunctures, blood pressure readings, and sun exposure), and using garments during air travel. More generally, any limb modification might be a potential cause of swelling and, thus, must be carefully reported to the clinician [10,11].

Analyzing more in detail specific therapeutic approaches, manual lymphatic drainage (MLD), compression therapy (CT), and the combination of the two (also called complete decongestive therapy, CDT) seem to be the most globally used [12,13,14,15]. More specifically, this approach can be divided into two steps: an early one, the decongestive phase, focused on the upper limb’s volume reduction, in which MLD and CT with multilayered bandaging are usually applied, and a late step, aimed at maintaining the current volume through compression garments and self-MLD [10]. However, the efficacy of MLD, CT, and CDT remains unclear, especially in more severe cases of BCRL [13,14]. Moreover, reconstructive microsurgical methods like lymphovenous anastomosis and vascular lymph node transfer are increasingly considered for patients, alongside liposuction-based procedures to address fatty fibrosis formation due to chronic lymphedema [16,17,18]. However, long-term adherence to self-management practices remains challenging, and the absence of consensus on diagnosis and measurement hinders outcome comparisons. No pharmacological strategies have demonstrated success in this regard [18].

Considering the heavy and constantly increasing disease burden, risk factors for the development of BCRL after BC treatment have been widely studied and described in the literature [3,10,19,20,21]. Over the years, axillary lymph node dissection (ALND) has been identified as the factor most related to BCRL: in fact, there is a 13 to 60% chance of presenting BCRL after ALND. The recent retrospective study conducted by Liu et al. notably identified that dissection of interpectoral (Rotter’s) lymph nodes (IPNs) is linked explicitly to severe breast cancer-related lymphedema [22]. When it is not mandatory, however, nowadays ALND has been substituted with sentinel lymph node biopsy (SLNB). This less traumatic technique leads to a four-times-lower risk of presenting BCRL [15].

Another well-known risk factor for BCRL is body mass index (BMI): higher values of BMI are related to higher BCRL incidence [23]. More specifically, obese patients with a BMI > 30 are 3.6 times more likely to develop BCRL within 6 months [24,25]. Another important independent risk factor is regional lymph node radiation (RLNR): this therapy alone, although very effective on tumor spreading and relapse, increases the risk of BCRL in both SLNB- and ALND-treated patients, at 3% and 9%, respectively [13,15,26]. Other risk factors, such as cellulitis, low-level arm volume changes after breast cancer surgery, lack of breast reconstruction, and postoperative axillary infection, hematoma, or seroma, are described in the literature [10,20,22,23,27,28,29,30].

Lastly, comorbidities and chronic medical treatments have been recently investigated as possible BCRL risk factors. Among comorbidities, hypertension represents the most studied one, followed by diabetes mellitus (DM), with both of them presenting controversial results [24,25,26,31]. Renal and chronic heart failure, liver disease, and collagen disease are still poorly evaluated, even though a recent Japanese nationwide study tried to determine their role as BCRL risk factors and found no statistical significance [32]. Considering commonly prescribed drugs, just a couple of studies investigating their role as potential risk factors for BCRL are available in the literature to date [33,34].

Given the known impact of fluid balance on lymphedema pathophysiology, we selected four widely prescribed drug classes (antihypertensives, NSAIDs, steroids, and hypoglycemics) based on their potential to influence fluid retention, capillary permeability, and inflammatory responses. Antihypertensive medications, particularly calcium channel blockers, can induce peripheral edema by increasing capillary hydrostatic pressure, while NSAIDs and steroids contribute to fluid retention through renal sodium retention mechanisms [35]. Additionally, steroids exert immunomodulatory effects that may alter lymphatic function, and certain hypoglycemic agents, such as thiazolidinediones, have been associated with increased interstitial fluid accumulation [36,37]. Despite their widespread use, the impact of these medications on BCRL remains largely unexplored. Investigating their potential contribution to BCRL pathogenesis may provide valuable insights for risk stratification and clinical management in breast cancer survivors.

Understanding the risk factors for BCRL plays a pivotal role in preserving the well-being of these patients, allowing clinicians to intercept patients at higher risk early and offer them preventive or more aggressive treatments. Therefore, due to the lack of literature on specific patient-related risk factors for developing BCRL, we first aimed to investigate whether commonly-prescribed pharmacological chronic treatments (specifically non-steroid anti-inflammatory drugs, steroids, anti-hypertensives, and hypoglycemics) might be involved in this pathological process. Secondly, we also evaluated the correlations with other well-known risk factors for BCRL in a European population of surgically treated BC survivors.

## 2. Materials and Methods

We conducted an observational retrospective cohort study to identify the risk factors for BCRL following BC treatment. To conform to the paper’s structure, we followed the STROBE checklist for observational studies.

Data were collected between January 2021 and April 2023 by consulting the medical records of a cohort of BC post-surgical survivors referring to the oncological rehabilitation outpatient service of a university hospital that serves a broad population from both urban and rural areas within one of the most densely populated regions of Northern Italy, ensuring access to rehabilitation services for a diverse range of patients following breast cancer surgery.

The inclusion criteria were as follows: female, age ≥ 18 years, previous BC treatment. The exclusion criteria were diagnosis of lymphedema that occurred prior to BC treatment, concurrent malignant tumors, incomplete wound healing, acute vascular disease (i.e., thrombophlebitis), skin problems (i.e., infection).

Medical records of the enrolled patients were then consulted by physical medicine and rehabilitation physicians to retrieve the following data: patient’s demographic and anthropometric characteristics (including age, weight, and height), tumor site and biological characteristics, BC treatment information (including date of surgery; type of breast surgery, i.e., lumpectomy, quadrantectomy, or mastectomy; axillary lymph node dissection; breast reconstruction; radiotherapy; chemotherapy and its timing; hormone therapy), breast cancer-related lymphedema diagnosis, and chronic pharmacological treatments (started before BC treatment, including NSAIDs, steroids, anti-hypertensives, and hypoglycemics).

The primary outcome measure of this study was the clinical assessment of BCRL as a secondary, progressive, and chronic condition characterized by an increase in the upper extremity or trunk swelling. Thus, the diagnosis was assessed by PM&R physicians through an accurate clinical evaluation and objective measures (≥2 cm increase in circumferential girth measurements).

More in detail, lymphedema is a clinical condition characterized by an abnormal protein-rich fluid buildup in the affected area, leading to swelling and potentially chronic inflammation, with or without fibrosis. This condition arises primarily due to malformation, underdevelopment, or disruption of the lymphatic circulation. It is typically classified as primary or acquired (secondary) and as acute or chronic. In breast cancer patients, arm lymphedema (also known as breast cancer-related lymphedema) is acquired, resulting from the removal or damaging of axillary lymph nodes and/or lymph vessels through therapeutic interventions, particularly after procedures such as axillary lymph node dissection (ALND) [38]. ALND is a surgical procedure aimed at removing lymph nodes located in the armpit area. Typically, it involves the extraction of nodes from levels I and II, possibly including level III nodes if cancer has spread to the lymph nodes or if larger nodes are detected through imaging tests. Level-one nodes are situated laterally to the pectoralis minor muscle, level-two nodes are found deep in the pectoralis minor muscle, and level-three nodes are located medially to the pectoralis minor muscle [39].

In determining the sample size for our retrospective study on risk factors for breast cancer-related lymphedema (BCRL), we based our calculations on existing literature. Specifically, considering the prevalence reported by Ren Y. et al. [40], where BCRL was found to be 6.8%, and utilizing a standard significance level (alpha error) of 0.05 and power (beta) of 0.80, we employed the formula for sample size estimation. The medium effect size (d = 0.5) was chosen to ensure a meaningful practical impact. The resulting calculated sample size was approximately 98, indicating the minimum number of subjects required for our study to detect significant differences with the specified confidence level and power.

### Statistical Analysis

Descriptive statistics were calculated to summarize the patient’s demographic, clinical, and treatment characteristics.

Absolute frequencies and percentages were reported for categorical variables, while means and corresponding standard deviations were reported for numerical ones, overall and stratified by the presence of BCRL.

Univariable and multivariable Cox regression models were applied to estimate the hazard ratios (HRs) and corresponding 95% confidence intervals (95% CIs) for the association between patients’ characteristics and the risk of BCRL. The stepwise method selected the variables included in the multivariable model considering a *p*-value threshold of 0.2 for entry and 0.1 for stay.

Statistical analyses were performed using SAS version 9.4 (SAS Institute, Cary, NC, USA).

## 3. Results

Initially, a total of 231 patients were first screened for eligibility, and 162 women met our inclusion criteria and were included in the study, with a mean age of 58.69 ± 13.73 years, a mean BMI of 24.77 kg/m^2^ (SD 4.73), and a 53% incidence of BCRL (BCRL group n = 86). More in detail, as shown in Table 1, the mean age was 61.72 years (SD 13.04) in the BCRL group and 55.30 years (SD 13.76) in the non-BCRL group. The BMI was 26.14 kg/m^2^ (SD 5.06) in the BCRL group and 23.23 kg/m^2^ (SD 3.81) in the non-BCRL group.

Regarding the association between patients’ characteristics and the risk of developing BCRL, we found that among demographic and anthropometric measures, BMI was associated with an increased risk of lymphedema (HR 1.07, 95% CI 1.02–1.11) while increasing height led to a decrease in lymphedema risk (HR: 0.95, 95% CI 0.92–0.98).

Moreover, chronic pharmacological medications and BC treatment of the enrolled patients are fully described in Table 2. In summary, the most frequently encountered chronic pharmacological therapy class in our cohort of patients was antihypertensives (49 patients, 30%), and the most frequent breast cancer treatments were axillary lymph node dissection (102, 63%), mastectomy (109, 68%), chemotherapy (103, 69%), and hormone therapy (99, 70%).

Concerning chronic pharmacological medications and breast cancer treatment, the implantation of a breast prosthesis was associated with a 75% increased risk of lymphedema compared to patients who did not undergo prosthesis implantation (HR 1.75, 95% CI: 1.09–2.80). Moreover, higher creatinine serum levels were associated with a decreased risk of lymphedema (HR 0.06, 95% CI: 0.01–0.52), as was axillary lymph node dissection (HR 0.35, 95% CI: 0.18–0.69). Other variables did not show a statistically significant association with BCRL.

The results of the multivariable model support the role of overweight and breast prosthesis in the increase in lymphedema and the protective role of creatinine, as shown in Table 3. Moreover, hypertension and radiotherapy seemed to increase the risk of lymphedema by 5.09 and 3.67 times, respectively, compared to non-hypertensive patients and those who did not undergo radiotherapy.

## 4. Discussion

In this retrospective cohort study, we found that various risk factors, including overweight (BMI ≥ 25), hypertension, breast prosthesis, and radiotherapy, were statistically related to BCRL development.

Our findings are consistent with recent literature, as corroborated by a recent umbrella review that underscored a statistically significant correlation between 14 factors and the development of BCRL. These factors comprise BMI, hypertension, tumor stage, pathologic T classification, ALND, level of ALND, number of LNs dissected, number of positive LNs, postoperative complications, postoperative infection, subcutaneous effusion, chemotherapy, radiotherapy, and breast reconstruction [21].

In detail, our univariate analysis showed that among demographic and anthropometric measures, smaller height and greater BMI at BCRL diagnosis were associated with an increased risk of lymphedema.

In the univariable analysis, a higher BMI was associated with an increased risk of BCRL. In the multivariable model, being overweight (BMI ≥ 25) also correlated with a higher risk of developing BCRL. Among studies supporting these data, Martínez-Jaimez P. et al. concluded that BMI was the only patient-related factor associated with increased risk [24]. Ugur et al. showed overweight to be the most crucial patient-related risk factor for breast cancer-related lymphedema [41]. Moreover, a higher BMI was stated to be associated with a more significant increase in BCRL risk by Kwan et al. [42]. Furthermore, Koelmeyer et al. found that a body mass index >30 increased the risk of BCRL [25]. Finally, Shen et al. demonstrated that higher body mass index and weight increase were risk factors for BCRL [26]. Moreover, the inverse association between height and BCRL risk in our univariate analysis may be explained by its relationship with BMI, as taller individuals with the same weight had a lower BMI, which is a well-established risk factor for lymphedema. Since BMI accounts for both height and weight, it is possible that the observed protective effect of height reflects the influence of body composition rather than an independent physiological mechanism. However, as height was not retained as a significant factor in the multivariable model, further research is needed to clarify its role in BCRL development.

Subsequently, we investigated the association between some commonly prescribed chronic pharmacological treatments and the development of BCRL. We intended to investigate that because numerous medications have been identified to induce or worsen fluid retention and tissue swelling through different mechanisms, including sodium overload, renal dysfunction, and vascular hyperpermeability, and through the disruption to the delicate balance between transcapillary flow, edema safety factors, and the capacity for lymphatic drainage. For example, calcium channel blockers can impede the pumping function of the collecting lymphatic vessels, potentially exacerbating swelling in individuals with lymphedema. Among medications causing fluid retention are corticosteroids, antihypertensives (i.e., calcium channel blockers, minoxidil, methyldopa, hydralazine, clonidine, and beta blockers), nonsteroidal anti-inflammatory drugs, and hypoglycemics (thiazolidinediones) [35,36,37,43].

More in detail, focusing specifically on breast cancer-related lymphedema, Stolarz et al. conducted a nested case-control study on a total of 717 cases and 1681 matched controls, and they concluded that the utilization of calcium channel blockers (CCBs) exhibited a significant association with the occurrence of lymphedema in breast cancer patients [34]. Conversely, the findings of Meijer et al. indicated that the use of anti-inflammatory drugs, anti-hypertensive drugs, and hormonal therapy within the first year after surgery does not elevate the risk of developing breast cancer-related lymphedema (BCRL) or worsen the severity of lymphedema in breast cancer patients [33]. In our cohort, univariate analysis did not reveal any significant associations between chronic pharmacological treatments and the risk of BCRL. Similarly, in the multivariable model, no medications, including antihypertensives, NSAIDs, corticosteroids, or hypoglycemic drugs, were independently associated with BCRL development. These findings suggest that previously reported associations between certain medications and lymphedema may have been influenced by underlying patient characteristics rather than a direct pharmacological effect.

Our multivariable analysis identified hypertension as a strong independent risk factor for BCRL, with hypertensive patients exhibiting more than a fivefold increased risk compared to non-hypertensive individuals (HR 5.09, 95% CI 1.88–13.79). This finding aligns with previous research suggesting a link between hypertension and BCRL, as reported by Shen et al. [26] and by Zhu et al. [44]. Chronic hypertension has been associated with vascular remodeling and impaired lymphatic function, potentially contributing to fluid accumulation and lymphedema development [26]. Increased blood pressure can compromise lymphatic vessel contractility, reducing their ability to effectively drain interstitial fluid and leading to tissue swelling [45]. Furthermore, hypertension is linked to systemic inflammation and endothelial dysfunction, which may impair lymphangiogenesis and exacerbate lymphatic congestion, increasing the risk of lymphedema in susceptible individuals [46]. While some studies have debated whether antihypertensive medications contribute to BCRL risk [33,34], our results suggest that the underlying hypertensive state itself, rather than its pharmacological management, is the primary driver of this association. Nevertheless, we acknowledge the challenge of disentangling the effects of hypertension from those of its pharmacological management, as comorbidities and medication use may be partially or fully overlapping. These findings highlight the importance of monitoring hypertensive patients for early signs of BCRL and considering blood pressure control as a potential factor in lymphedema prevention strategies. Future prospective studies are needed to explore the mechanisms linking hypertension to BCRL and to evaluate whether specific antihypertensive regimens influence lymphedema risk. In the univariate analysis, the only factor significantly associated with an increased risk of breast cancer-related lymphedema was the implantation of a breast prosthesis (HR: 1.75; 95% CI: 1.09–2.80). However, in the multivariate analysis, both breast prosthesis implantation (HR: 8.93; 95% CI: 2.77–28.81) and radiotherapy (HR: 3.67; 95% CI: 1.43–9.38) were identified as independent risk factors for lymphedema development.

Taxan-based chemotherapy, regional lymph node radiation therapy, and ALND already represent well-established risk factors for BCRL, as the most recent review showed [26,31,33,34]. The magnitude of axillary surgery and radiation therapy are undoubtedly notable risk factors.

Finally, several studies have investigated the relationship between breast prosthesis implantation and the risk of breast cancer-related lymphedema (BCRL), yielding mixed results. Some research suggests that implant-based reconstruction may increase the risk of BCRL [47], possibly due to additional surgical trauma and compromised lymphatic drainage. However, some studies suggest that prosthesis implantation may not increase the risk of lymphedema and could even have a protective effect [48,49,50]. These discrepancies highlight the need for further research to clarify the impact of breast prosthesis implantation on BCRL risk.

Unexpectedly, in our univariable analysis, ALND was associated with a lower risk of BCRL (HR 0.35, 95% CI: 0.18–0.69). However, this finding may reflect selection bias: for instance, patients undergoing ALND might have received more intensive follow-up, leading to earlier diagnosis and management of lymphedema, or there may be unmeasured factors differentiating the groups. Moreover, in our multivariable analysis, ALND was no longer an independent risk factor, suggesting that the apparent protective effect observed in the univariable analysis was mediated by variables such as BMI, hypertension, breast prosthesis implantation, and radiotherapy. Prospective studies are needed to further explore these aspects.

Moreover, in our univariable analysis, higher creatinine levels were statistically associated with a lower risk of BCRL (HR 0.06, 95% CI: 0.01–0.52). Nevertheless, this result requires careful interpretation, as the mean creatinine values in both groups (0.65 mg/dL in BCRL patients vs. 0.71 mg/dL in non-BCRL patients) remained well within the normal physiological range for women. Even considering the maximum observed values (0.99 mg/dL in BCRL patients vs. 1.12 mg/dL in non-BCRL patients), the difference is minimal and does not suggest any clinically meaningful variation. Although statistically significant, this finding is unlikely to reflect a true protective effect, as no clear physiological mechanism links higher creatinine levels to a reduced risk of BCRL. It is therefore possible that this association is due to random variability in the data rather than a biologically relevant relationship. Further research is needed to clarify this aspect.

To the best of our knowledge, this is one of the first few studies searching for an association between commonly prescribed chronic drugs and the development of BCRL, thus representing a pioneer investigation in this field. However, our study has some limitations. Firstly, the retrospective monocentric design could hinder any robust conclusion about the results obtained. Moreover, the potential overlap between comorbidities and medication use makes it challenging to determine whether the increased BCRL risk is attributable to the underlying condition (e.g., hypertension) or its treatment. Due to the retrospective nature of our study and the available data, we were unable to disentangle these effects, which represents an additional limitation. Secondly, the BCRL incidence rate in our cohort may be higher than expected due to a sample bias, because we enrolled patients already referred to our rehabilitation service, rather than enrolling all BC survivors. While all patients undergoing quadrantectomy or mastectomy are routinely referred, it is possible that those with fewer comorbidities or less severe symptoms may not attend, potentially leading to an overrepresentation of patients with higher risk for BCRL in our cohort. Thirdly, the small sample size and the small numerosity of each subpopulation prevented us from conducting a more detailed study of the pharmacological treatments. Specifically, our limited sample size did not allow for stratification by antihypertensive drug class, which could have provided valuable insights into potential differential effects of specific medications, such as beta-blockers versus calcium channel blockers, on BCRL risk. The low number of patients using each drug class may have reduced the statistical power, potentially masking associations between medication use and BCRL development. Furthermore, our sample size estimation was based on BCRL prevalence reported in the literature, which may not fully account for differences in population characteristics or risk factor distribution. Future studies with larger and more diverse cohorts are needed to further validate these findings. Additionally, factors such as physical activity, diet, and hydration status, which were not adjusted for in our study, may have acted as confounders. These lifestyle factors could influence fluid retention and lymphatic function, and their absence as variables may have affected the findings related to pharmacological treatments. This constraint highlights the need for larger, multicentric prospective studies to validate our findings and further investigate the impact of commonly prescribed medications on BCRL. In conclusion, socioeconomic and lifestyle factors, such as educational level, employment status, and smoking habits, were not systematically collected in our database. These variables could potentially influence BCRL risk and should be considered in future prospective studies to provide a more comprehensive understanding of the factors contributing to its development.

Nonetheless, the present study is a starting point for future research.

## 5. Conclusions

This retrospective cohort study identified breast prosthesis implantation, radiotherapy, hypertension, and overweight (BMI > 25) as independent risk factors for BCRL in the multivariate analysis. While radiotherapy and overweight are well-established risk factors, our findings suggest that hypertension and breast prosthesis implantation may also play a significant role in BCRL development. These results highlight the need for further research to better understand the potential mechanisms linking hypertension and breast prosthesis implantation to lymphatic dysfunction, as well as to explore preventive strategies for high-risk patients.

## Figures and Tables

**Table 1:** Descriptive statistics of patients’ demographic and anthropometric characteristics overall and by lymphedema status, and the hazard ratios (HRs) and corresponding 95% confidence intervals (95% CIs) for the association between patients’ characteristics and risk of lymphedema derived from the univariable model.

	Lymphedema		Total	Missing	HR (95% CI)
	No	Yes			
	N = 76	N = 86	162		
	Mean (SD)	Mean (SD)	Mean (SD)		
Age (years)	55.3 (13.76)	61.72 (13.04)	58.69 (13.73)	1	0.99 (0.98–1.01)
Height (cm)	162.43 (6.54)	158.52 (6.34)	160.36 (6.71)	0	0.95 (0.92–0.98)
Weight (kg)	61.29 (10.52)	65.55 (12.3)	63.55 (11.66)	0	1.01 (0.99–1.03)
BMI	23.23 (3.81)	26.14 (5.06)	24.77 (4.73)	0	1.07 (1.02–1.11)
	N (%)	N (%)	N (%)		
Overweight (BMI > 25)					
No	59 (77.63)	41 (47.67)	100 (61.73)	0	1
Yes	17 (22.37)	45 (52.33)	62 (38.27)		1.46 (0.95–2.25)

**Table 2:** Descriptive statistics of patients’ chronic pharmacological medications and BC treatment, overall and by lymphedema status, and the hazard ratios (HRs) and corresponding 95% confidence intervals (95% CIs) for the association between patients’ characteristics and risk of lymphedema derived from the univariable model.

	Lymphedema		Total	Missing	HR (95% CI)
	No	Yes			
	N = 76	N = 86	N = 162		
	Mean (SD)	Mean (SD)	Mean (SD)		
Creatinine	0.71 (0.16)	0.65 (0.17)	0.69 (0.16)	52	0.06 (0.01–0.52)
	N (%)	N (%)	N (%)		
Antidiabetics					
No	75 (98.68)	75 (87.21)	150 (92.59)	0	1
Yes	1 (1.32)	11 (12.79)	12 (7.41)		1.31 (0.68–2.54)
Antihypertensives					
No	62 (81.58)	51 (59.3)	113 (69.75)	0	1
Yes	14 (18.42)	35 (40.7)	49 (30.25)		1.15 (0.74–1.78)
Hypertension					
No	59 (77.63)	48 (55.81)	107 (66.05)	0	1
Yes	17 (22.37)	38 (44.19)	55 (33.95)		1.27 (0.82–1.96)
NSAIDs					
No	76 (100)	80 (93.02)	156 (96.3)	0	1
Yes	0 (0.00)	6 (6.98)	6 (3.7)		1.49 (0.64–3.45)
Steroids					
No	71 (93.42)	83 (96.51)	154 (95.06)	0	1
Yes	5 (6.58)	3 (3.49)	8 (4.94)		1.36 (0.42–4.36)
ALND					
No	26 (34.21)	76 (88.37)	102 (62.96)	0	1
Yes	50 (65.79)	10 (11.63)	60 (37.04)		0.35 (0.18–0.69)
Intervention type					
Quadrantectomy	19 (25)	33 (38.82)	52 (32.3)	1	1
Mastectomy	57 (75)	52 (61.18)	109 (67.7)		1.28 (0.81–2.01)
Breast prosthesis					
Not implanted	27 (35.53)	44 (51.16)	71 (43.83)	0	1
Implanted	49 (64.47)	42 (48.84)	91 (56.17)		1.75 (1.09–2.80)
Chemotherapy					
No	32 (42.67)	15 (20)	47 (31.33)	13	1
Yes	43 (57.33)	60 (80)	103 (68.67)		1.45 (0.82–2.57)
Hormone therapy					
No	25 (37.31)	17 (22.97)	42 (29.79)	22	1
Yes	42 (62.69)	57 (77.03)	99 (70.21)		1.14 (0.66–1.97)
Radiotherapy					
No	49 (64.47)	15 (20.83)	64 (43.24)	14	1
Yes	27 (35.53)	57 (79.17)	84 (56.76)		1.55 (0.86–2.80)
Chemotherapy timing					
Neoadjuvant	15 (34.09)	8 (13.79)	23 (22.55)	64	1
Adjuvant	20 (45.45)	40 (68.97)	60 (58.82)		1.07 (0.49–2.33)
Both	9 (20.45)	10 (17.24)	19 (18.63)		1.49 (0.59–3.80)

**Table 3:** Hazard ratios (HRs) and corresponding 95% confidence intervals (95% CIs) for the association between patients’ characteristics and the risk of lymphedema derived from the multivariable model.

	HR (95% CI)
Overweight (BMI ≥ 25)	
No	1
Yes	2.90 (1.18–7.14)
Creatinine	0.08 (0.01–0.86)
Hypertension	
No	1
Yes	5.09 (1.88–13.79)
Breast prosthesis	
Not implanted	1
Implanted	8.93 (2.77–28.81)
Radiotherapy	
No	1
Yes	3.67 (1.43–9.38)

## Data Availability

The data presented in this study are available on request from the corresponding author.
